# Equidade na atenção à saúde de mulheres no Haiti

**DOI:** 10.26633/RPSP.2017.34

**Published:** 2017-03-23

**Authors:** Nadège Jacques, Stela Nazareth Meneghel, Ian Meneghel Danilevicz, Joyce Mendes de Andrade Schramm, Alcindo Antônio Ferla

**Affiliations:** 1 Universidade Federal do Rio Grande do Sul (UFRGS) Programa de Pós-Graduação em Saúde Coletiva Porto Alegre (RS) Brasil Universidade Federal do Rio Grande do Sul (UFRGS), Programa de Pós-Graduação em Saúde Coletiva, Porto Alegre (RS), Brasil.; 2 Universidade Federal do Rio Grande do Sul (UFRGS) Programa de Pós-Graduação em Saúde Coletiva e Programa de Pós-Graduação em Enfermagem Porto Alegre (RS) Brasil Programa de Pós-Graduação em Saúde Coletiva e Programa de Pós-Graduação em Enfermagem, Porto Alegre (RS), Brasil.; 3 Universidade Federal de São Carlos (UFSCar) Pós-Graduação em Estatística São Carlos (SP) Brasil Universidade Federal de São Carlos (UFSCar), Pós-Graduação em Estatística, São Carlos (SP), Brasil.; 4 Fundação Instituto Oswaldo Cruz Escola Nacional de Saúde Pública Rio de Janeiro (RJ) Brasil Fundação Instituto Oswaldo Cruz, Escola Nacional de Saúde Pública, Rio de Janeiro (RJ), Brasil.

**Keywords:** Gênero e saúde, atenção primária à saúde, saúde da mulher, equidade em saúde, Haiti, Gender and health, primary health care, women’s health, health equity, Haiti

## Abstract

**Objetivos.:**

Descrever a atenção básica em saúde prestada às mulheres no Haiti e avaliar a equidade da atenção.

**Métodos.:**

Neste estudo transversal, 114 mulheres atendidas na atenção básica nos 10 departamentos de saúde do país foram entrevistadas. Para analisar a equidade, foram utilizados dois grupos de indicadores: acesso (tempo de caminhada para chegar aos serviços, tempo de espera na fila de consultas e necessidade de pagamento) e qualidade (conhecer o nome do prestador de serviços, tempo de consulta e discriminação). Pagamento e preconceito foram escolhidos como desfechos respectivamente para acesso e qualidade da atenção.

**Resultados.:**

*A maioria das mulheres tinha menos de 30 anos (59,0%), era negra (92,1%) e migrante (63,2%); apenas 21,3% souberam informar a renda familiar e 47,4% eram alfabetizadas. A maioria das consultas foi realizada em menos de 10 minutos (68,3%). O nome do profissional que prestou o serviço não era conhecido por 72,7% das mulheres. As consultas foram pagas por 63,4%, especialmente na região Sul* (P *= 0,016). Ainda, as mulheres no Sul levaram mais tempo para chegar aos serviços. Aquelas que não pagaram tiveram consultas com menor duração* (P *< 0,001). A discriminação nos serviços de saúde foi detectada em 28,9% das entrevistadas.*

**Conclusões.:**

Esta pesquisa mostrou dificuldades no acesso e discriminação na atenção primária em saúde prestada a mulheres no Haiti e indica o gênero como uma categoria de análise importante para avaliar a equidade nos serviços de saúde.

## Abstract (en)

***Objective*.:**

To describe primary health care provided to women in Haiti and evaluate equity in the care provided to this group.

***Methods*.:**

In this cross-sectional study, 114 women receiving primary health care services in the country’s 10 health departments were interviewed. Two groups of indicators were used for analysis of equity: access indicators (walking time to reach services, waiting time, and need to pay for the service) and quality (knowing the name of the healthcare provider, length of the consultation, and discrimination). Payment and discrimination were used as outcomes for access and quality, respectively.

***Results*.:**

Most women were younger than 30 years of age (59.0%), black (92.1%), and migrants (63.2%). Family income was known by only 21.3%, and 47,4% were literate. Most consultations lasted less than 10 minutes (68.3%). The provider’s name was not known by 72.7% of the women interviewed. Consultations were paid by 63.4%, especially in the South (*P* = 0.016). Also, women in the South spent more time walking to reach health services. Those who did not pay had shorter consultations (*P* < 0.001). Finally, discrimination was detected in 28.9% of the women interviewed.

**Conclusions.:**

This research revealed difficulties in the access to and discrimination in the care provided to women in Haiti, and suggests that gender is an important category of analysis to evaluate health equity.

Equidade é um conceito político, considerado um imperativo ético associado aos princípios de justiça social e direitos humanos. Pode-se tipificar a equidade como horizontal, quando há tratamento igual para indivíduos iguais, e vertical, quando ocorre tratamento desigual para indivíduos diferentes (1, 2).

O gênero é considerado a apropriação social do sexo biológico a partir de quatro elementos: representações simbólicas e culturais sobre cada um dos sexos; normas para definir o masculino e o feminino; binarismo dos sexos; e identidade sexual subjetiva pautada em prescrições normativas (3). Embora esteja implícito o aspecto relacional, o conceito pode ser empregado em referência a um só sexo.

A equidade em saúde pressupõe iguais oportunidades de acesso aos recursos disponíveis, política de saúde universal e distribuição democrática de poder (4). A equidade em relação à saúde das mulheres enfatiza as necessidades de cada etapa do ciclo vital, a qualidade dos serviços e os cuidados na vida sexual e reprodutiva. A equidade de gênero em saúde se refere às diferenças no acesso e às especificidades da atenção dirigida a homens e mulheres, mas também considera a adequação do cuidado de acordo com as diferenças de classe, raça/etnia, ocupação, geração e orientação sexual entre as próprias mulheres, visando atendê-las segundo suas necessidades e oferecer ações que possam diminuir as desigualdades injustas entre elas (5).

Na América Latina e Caribe, ao analisar a equidade na atenção à saúde das mulheres, observa-se que as pobres, as indígenas e as negras estão em desvantagem em relação ao acesso a serviços e atenção adequada. No Haiti, um dos países mais pobres dessa região, grande parte das famílias, principalmente as chefiadas por mulheres, vive em situação de insegurança alimentar, 78% da população sobrevive com menos de 2 dólares por dia (6) e o acesso aos bens essenciais, aos serviços de saúde, à justiça e à seguridade social é limitado (7).

A atual política nacional de saúde haitiana afirma garantir os princípios de universalidade, integralidade e equidade na atenção à saúde, fornecendo um pacote mínimo de serviços à população do país (8, 9). Contudo, o sistema de saúde haitiano apresenta graves problemas de funcionamento, de organização e de gestão, o que resulta em uma oferta de cuidados fragmentada, com acesso restrito e baixa qualidade. A cobertura da população não chega aos 60%, e os recursos humanos são insuficientes e têm baixa qualificação (10).

As políticas públicas dirigidas às mulheres se referem principalmente à saúde materna. Em 2008, foi lançado no Haiti um programa de cuidados obstétricos gratuitos, considerado porta de entrada ao sistema de saúde (11). Mesmo assim, a maioria dos partos ainda é domiciliar, a mortalidade materna continua sendo uma das mais elevadas do mundo (380/100 000 nascidos vivos) e apenas 18% das mulheres têm acesso a métodos contraceptivos modernos. Além disso, o país enfrenta o fenômeno da feminização da Aids, com taxas de 2,4% em mulheres contra 1,6% em homens; os programas de saúde não enfocam os direitos sexuais e reprodutivos e a política de saúde não inclui a perspectiva de gênero (12-15).

Frente a essa situação, o objetivo do presente estudo foi identificar como se dá a equidade no atendimento às mulheres na atenção primária em saúde no Haiti.

## MATERIAIS E MÉTODOS

Este estudo transversal foi realizado durante um curso de epidemiologia de serviços (16, 17) no âmbito da Cooperação Brasil-Cuba-Haiti, um projeto instituído com o objetivo de fortalecer o sistema de saúde e de vigilância epidemiológica no Haiti após o terremoto ocorrido em 2010 naquele país (18). O estudo faz parte de uma pesquisa brasileira sobre a equidade de gênero na atenção a mulheres como marcador de integralidade na atenção básica (19).

Os dados foram obtidos por meio de entrevistas realizadas com uma amostra intencional de usuárias de serviços de saúde no Haiti. As participantes foram escolhidas aleatoriamente enquanto aguardavam atendimento em salas de espera de centros de saúde localizados nas cidades-sede dos 10 departamentos sanitários do Haiti. Foi utilizado um questionário semiestruturado adaptado de um estudo brasileiro sobre saúde da mulher (20) que compreendeu 40 questões organizadas em quatro blocos: características demográficas e socioeconômicas, acesso geográfico e econômico, saúde sexual e reprodutiva e qualidade e aceitabilidade dos serviços. O questionário foi elaborado no Brasil e revisado no Haiti através de um grupo de discussão com os profissionais que fariam a coleta dos dados, excluindo algumas questões e incluindo outras consideradas mais adequadas à realidade do país.

O questionário foi traduzido para a língua crioula haitiana e testado em um piloto realizado em uma unidade de saúde localizada na cidade de Carrefour. Os entrevistadores eram profissionais de saúde que trabalhavam nas direções departamentais de saúde do Haiti e participavam do curso de epidemiologia e serviços. As entrevistas foram realizadas entre abril e julho de 2013. Foram ouvidas 114 mulheres em idade reprodutiva que usavam os serviços de atenção primária há pelo menos 1 ano. Para potencializar a análise dos dados, os 10 departamentos de saúde foram reagrupados em três grandes regiões: Centro (Centro, Artibonite e Oeste), Norte (Norte, Nordeste e Noroeste) e Sul (Sul, Sudeste, Nippes e Grand Anse), utilizando como critério a proximidade geográfica dos mesmos.

As variáveis demográficas, constantes do primeiro bloco e incluídas na análise, foram: alfabetização, migração, situação conjugal e região. Língua, raça e religião não foram usadas porque os dados eram muito homogêneos. A renda não foi utilizada porque havia um percentual muito grande de mulheres que não sabia qual o montante do ganho familiar. O bloco referente à vida sexual e reprodutiva não foi analisado neste estudo.

Para estudar a equidade na atenção à saúde de mulheres, utilizaram-se dois grupos de indicadores (20, 21), os de acesso e os de qualidade. O acesso, definido como a possibilidade de usar serviços cultural e economicamente viáveis, contemplou as dimensões de disponibilidade, localização geográfica e organização dos serviços, medidas pelos indicadores tempo de caminhada para chegar ao serviço de saúde e tempo de espera na fila, e a dimensão de custos, medida pelo pagamento ou não da consulta. Os indicadores selecionados para averiguar a qualidade de atenção referem-se ao acolhimento (saber o nome do profissional que prestou o serviço), à atenção adequada (duração da consulta) e ao respeito à usuária (ausência de discriminação) (21). Pagamento e preconceito foram escolhidos como desfechos respectivamente para acesso e qualidade da atenção, tendo sido realizados testes de associação entre si e com outras variáveis do estudo.

A variável discriminação foi constituída pela união das respostas “sim” e “não respondeu”, consideradas respectivamente como preconceito declarado e silenciado. A pergunta realizada para detectar discriminação foi: “Você sofreu algum preconceito ou foi maltratada no serviço de saúde em razão de sua religião, situação econômica, profissão, orientação sexual, hábitos ou pela doença? Conte como foi”. Ausência de discriminação foi considerada apenas quando houve uma declaração explícita por parte das mulheres de não terem sofrido discriminação.

A análise estatística foi realizada por meio do programa *Statistical Package for the Social Sciences* (SPSS, versão 19.0). Para testar as associações entre as variáveis qualitativas, foi utilizado o teste exato de Fisher, considerando significativos os valores de *P* < 0,05 (22). Na aplicação do teste de Fisher para variáveis exploratórias que possuem mais de duas opções, como região (Centro, Sul, Norte), analisaram-se os resíduos padronizados para identificar em que locais houve diferenças significativas. Como os resíduos são padronizados, os valores de referência encontram-se no intervalo de -2 a + 2; valores menores do que -2 ou maiores do que +2 são significativos.

Para analisar a variável quantitativa “tempo de caminhada para chegar ao serviço de saúde” utilizou-se a raiz quadrada dos valores estratificados pelas três macrorregiões do país, testados através da análise de variância (ANOVA). Depois da ANOVA, foi aplicado o teste de Tukey para averiguar em que regiões havia diferenças estatisticamente significativas.

Este projeto de pesquisa foi aprovado pelo Comitê de Ética em Pesquisa da Universidade Federal do Rio Grande do Sul (UFRGS, protocolo 188.515). Todos os aspectos éticos descritos na Resolução 466/12 (23) foram respeitados. Os objetivos da pesquisa foram explicados e as entrevistadas que aceitaram participar assinaram um termo de consentimento livre e esclarecido. Elas não foram identificadas nominalmente nos questionários, e foi-lhes assegurada a confidencialidade das informações.

## RESULTADOS

Os resultados apresentados se referem às 114 mulheres entrevistadas nos 10 departamentos sanitários do Haiti. A tabela 1 mostra as variáveis demográficas e as variáveis de acesso e qualidade da atenção.

Todas as mulheres eram haitianas. Como mostra a tabela 1, a maioria era negra (92,1%) e falante apenas da língua crioula haitiana (91,2%). A média de idade foi de 31 anos. Das 73 mulheres que referiram estar trabalhando, 44 (60,2%) eram vendedoras de rua, uma ocupação insegura e estigmatizada. Do total de 114 mulheres, 81 (71,1%) declararam de forma explícita não ter sofrido discriminação (tabela 1).

**TABELA 1.** Variáveis demográficas, de acesso e de qualidade, Haiti, 2013

Variáveis	No	%	Total
Demográficas			
Idade (média = 31 anos; DP = 11)			
< 30 anos	68	59,0	
≥ 30anos	46	41,0	114
Raça			
Negra	105	92,1	
Mestiça	7	7,1	112
Anos de estudo (media = 6,5 anos; DP= 4,8)			
Alfabetizadas	54	47,4	
Não alfabetizadas	32	28,1	86
Religião			
Protestante	61	53,5	
Outra	53	46,5	114
Língua			
Língua crioula haitiana	104	91,2	
Francês	10	8,8	114
Renda domiciliar (média informada = 4.727,32 gourdes, US$ 66,8)			
Sem ou ignorada	89	78,7	
Informada	24	21,3	113
Trabalha			
Sim (44 = vendedoras de rua/ambulantes)	73	65,2
Não	39	34,8	112
Estado conjugal			
Solteira/sem companheiro	53	50,5	
Casada	52	49,5	105
Filhos (média = 2,6)			
Sem filhos	23	20,2	
Com filhos	91	79,8	114
Migração			
Sim	72	63,2
Não	42	36,8	114
Violencia			
Sim	49	44,5
Não	61	55,5	110
Região onde mora			
Norte	28	25,3	
Sul	40	36,0	
Centro	43	38,7	
Variáveis de acesso			
Tempo para chegar no serviço de saúde (média = 68 min; DP = 71 min)			
Tempo na fila de espera			
< 1h	70	66,0	114
> 1h	36	34,0	106
Pagamento			
Sim	71	63,4	
Não	41	36,6	112
Variáveis de qualidade			
Sabe o nome do profissional			
Sim	30	27,3	
Não	80	72,7	110
Duração da consulta			
< 10 min	71	68,	
> 10 min	33	31,7	104
Discriminação institucional
Sim	10	8,8	
Não	81	71,1	
Sem resposta	23	20,1	

A tabela 2 mostra que, embora a maioria das mulheres entrevistadas tenha pagado pelos serviços de saúde recebidos, na região Sul houve maior proporção de pagantes (*P* = 0,016). A análise dos resíduos padronizados indica que essa diferença se dá principalmente entre o Sul e o Norte. A relação entre pagamento e tempo de espera na fila foi paradoxal. O pagamento não garantiu atendimento mais rápido – de fato, as mulheres que não pagaram foram atendidas mais rapidamente (*P* = 0,014). A duração das consultas foi curta para todas, mas as que não pagaram foram atendidas em menos tempo (P < 0,001). A maioria das mulheres não sabia o nome do profissional que as atendeu e o pagamento não fez diferença em relação ao conhecimento do nome do prestador de serviço (*P =* 0,067). Em relação à existência de discriminação ou preconceito (tabela 3), 28,9% sofreram discriminação nos serviços (preconceito declarado ou silenciado). As mulheres da região central, que inclui a capital do país, Porto Príncipe, declararam ter sofrido mais discriminação (*P* < 0,001). A associação entre região e preconceito foi significativa, e a análise padronizada dos resíduos evidenciou que o centro do país foi a região na qual as mulheres mais relataram preconceito. Usuárias que pagaram a consulta foram um pouco menos discriminadas do que as demais, porèm a significância foi limítrofe (*P* = 0,071).

**TABELA 2.** Relação entre pagamento da consulta e variáveis do estudo, Haiti, 2013

	Pagamento da consulta				
	Sim	%	Não	%	*P*^a^
Migração					
Sim	46	64,8	24	58,5	0,323
Não	25	35,2	17	41,5	
Alfabetização					
Sim	30	55,5	23	74,1	0,069
Não	24	44,5	8	25,9	
Região					
Centro	27	38,6	16	39,0	0.016
Norte	12	17,1	16	39,0	
Sul	31	44,3	9	22,0	
Fila de espera					
< 1 h	38	57,6	32	80,0	0,014
> 1 h	28	42.4	8	20,0	
Duração da consulta					
< 10 min	35	55,6	35	89,7	< 0,001
> 10 min	28	44,4	4	10,3	
Nome do profissional					
Sabe	15	22,1	15	37,5	0,067
Não	53	77,9	25	62,5	

a Teste exato de Fisher unilateral (*P* < 0,05).

**FIGURA 1.**
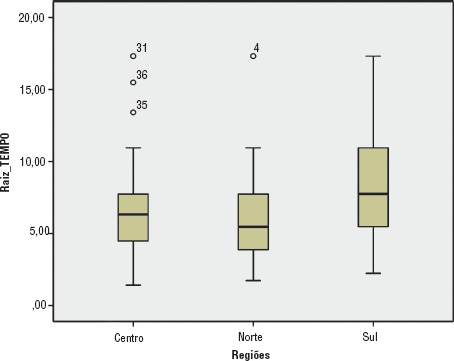
Tempo de caminhada em minutos para chegar ao serviço de saúde segundo região, Haiti, 2013^a^

^a^ As posições 31, 34, 35 e 4 são outlyers pela técnica de boxplot, porém nenhuma delas foi retirada da análise de variância (ANOVA). Caso fossem removidas, a diferença entre Sul e demais regiões seria ainda maior, visto que todos os outlyers pertencem ao Centro e Norte e são maiores do que a mediana, ou seja, a sua permanência abranda a diferença entre regiões em relação ao tempo de caminhada para chegar a um serviço de saúde.

O tempo médio despendido para as mulheres chegarem aos serviços de saúde caminhando nas três regiões do país (figura 1) foi de 48,1 minutos no Centro, 37,9 minutos no Norte e 73,8 minutos no Sul. A comparação entre as regiões foi realizada usando o teste ANOVA (P = 0,027) e após o teste de Tukey, que permitiu observar que as mulheres do Sul caminham mais em relação ao Norte (P = 0,031) e em relação ao Centro (P = 0,084), onde está a capital e há mais serviços, embora o nível de significância do teste tenha sido limítrofe. Mesmo assim, resultados com P até 0,10 são aceitáveis para estudos do campo das ciências sociais e saúde (24).

**TABELA 3.** Relação entre preconceito e variáveis do estudo, Haiti, 2013

	Preconceito				
	Sim	%	Não	%	P
Alfabetização					
Sim	18	69,2	36	60,0	0,286
Não	8	30,8	24	40,0
Migração					
Sim	19	57,6	53	65,4	0,281
Não	14	42,4	28	34,6
Fila de espera					
< 1 h	21	70,0	51	65,4	0,414
> 1 h	9	30,0	27	34,6
Pagamento					
Sim	17	51,5	54	68,4	0,071
Não	16	48,5	25	31,6
Duração consulta					
< 10 min	24	80,0	47	63,5	0,078
> 10 min	6	20,0	27	35,5
Nome do profissional					
Sim	8	25,0	22	28,2	0,463
Não	24	75,0	56	71,8
Região					
Centro	28	87,5	15	18,5	**< 0,001**
Norte	3	9,4	27	33,3
Sul	1	3,1	39	48,1	
Violência					
Sim	14	43,8	38	46,9	0,463
Não	18	56,3	43	53,1	

a Teste exato de Fisher unilateral (*P* < 0,05).

## DISCUSSÃO

Apesar de muitos dos países latinoamericanos e caribenhos terem elaborado legislações e políticas focadas nas mulheres, grande parte dos serviços de saúde ainda desconsidera a equidade de gênero na atenção às usuárias. Embora as mulheres utilizem mais os serviços de saúde do que os homens, elas continuam morrendo por causas evitáveis e, em vários países, inclusive o Haiti, onde menos de 50% dos partos são realizados em condições adequadas (60% na capital, 56% em zonas urbanas e 23% em regiões rurais), há elevado risco de morte materna (25, 26). As taxas de desemprego femininas são elevadas e os salários são menores do que os masculinos, agudizando a pobreza feminina e as más condições de saúde (27, 28). Os atendimentos em saúde são massificados e não se consideram as especificidades de idade, ocupação e orientação sexual.

A pesquisa realizada no Haiti mostrou a existência de numerosos obstáculos ao acesso e à qualidade dos cuidados dispensados às usuárias na atenção primária de saúde. As mulheres despenderam muito tempo caminhando para chegar aos serviços, que eram, em sua maioria, pagos. O tempo de duração das consultas foi curto e as mulheres não sabiam o nome das pessoas que as tinham atendido, mostrando a falta de diálogo entre profissionais de saúde e pacientes. Muitas silenciaram quando perguntadas acerca de preconceito, possivelmente pormedo de serem discriminadas se retornassem ao serviço de saúde. Além do mais, deve-se considerar que, apesar de tudo, as mulheres entrevistadas faziam parte da metade da população que consegue chegar aos serviços de saúde e ser atendida (8, 10), ou seja, estão em melhor situação do que outras tantas que sequer chegam aos serviços.

Embora o acesso geográfico seja apenas uma das dimensões da equidade no setor saúde, o tempo médio de caminhada para chegar a um serviço de saúde não deve passar de 20 a 30 minutos (29). Esse tempo é maior nas regiões rurais, onde vive 50% da população (12) e onde as estradas são precárias e os serviços dispersos. A enquete sobre a mortalidade, morbidade e uso dos serviços (EMMUS) realizada a cada 5 anos no Haiti (a primeira em 1987, a segunda em 1994-1995, a terceira em 2000, a quarta em 2005-2006 e a quinta em 2012) mostrou que 43% dos entrevistados referiram a distância como um entrave para o acesso aos serviços de saúde (26).

O pagamento direto dos serviços pelos usuários é outra restrição para a entrada e uso do sistema de saúde haitiano. A maioria das mulheres entrevistadas havia realizado algum tipo de pagamento. Embora a Politica Nacional de Saúde (9) assegure a universalidade e a gratuidade dos cuidados, uma grande parte dos serviços públicos e organizações não governamentais cobra taxas. As usuárias atendidas gratuitamente tiveram consultas mais rápidas, porém as pessoas que pagam são muitas vezes as mais vulneráveis, pela falta de acesso aos serviços públicos. Os resultados da EMMUS-IV referentes aos anos de 2005 a 2006 evidenciaram que 41% dos doentes não chegaram aos serviços de saúde devido aos custos elevados (30). O pagamento constitui uma barreira ao acesso, mesmo quando as taxas são simbólicas, e impacta negativamente o orçamento familiar (31). Os dados do presente estudo mostraram uma precariedade na atenção à saúde que atinge a todas; assim, não houve diferença entre as mulheres que pagaram ou não quanto ao tempo na fila de espera, e a maioria delas não sabia o nome do profissional que as atendeu. Essa distância entre profissionais e usuárias pode ocorrer pela exiguidade de tempo na consulta ou pela desigualdade social entre profissionais e usuárias. Esse indicador aponta para relações despersonalizadas entre profissionais e usuárias, dificultando o acolhimento, a escuta e a construção do vínculo (21). Não há consenso acerca do tempo de duração apropriado para uma consulta médica, porém pode-se considerar que o tempo é insuficiente quando impossibilita as pessoas de serem ouvidas e expressarem suas demandas (32), o que parece ter ocorrido na amostra estudada, em que grande parte das consultas durou menos de 10 minutos.

A qualidade e a aceitabilidade dos serviços significa que os usuários confiam que o profissional agirá em seu interesse, demonstrando respeito, sem manifestar preconceitos e discriminação, independentemente das características das pessoas atendidas (21). Quase 10% das mulheres entrevistadas afirmaram ter sofrido discriminação por parte dos profissionais nos serviços de saúde, enquanto 20% mantiveram-se em silêncio frente a essa pergunta (33). Neste estudo, consideramos o silêncio como afirmação muda frente aos preconceitos e à discriminação ocorridos nos serviços de saúde. As mulheres podem ter mantido silêncio porque precisam seguir frequentando o serviço e temem ser mal recebidas se expressarem críticas.

A prevalência referida de violências (física, psicológica, sexual ou econômica) em algum momento da vida foi de 44,5%, enquanto que outros estudos com mulheres haitianas reportaram prevalências em torno de 25% (26, 34). Essa prevalência elevada pode estar associada ao padrão patriarcal da sociedade haitiana e indica a necessidade de os serviços de saúde incluírem essa questão no atendimento às mulheres na atenção primária.

### Considerações finais

A equidade na atenção de mulheres na atenção primária ainda constitui uma perspectiva pouco utilizada para o acompanhamento de serviços, inclusive no Haiti, onde esse tema é pouco abordado. Os resultados deste estudo evidenciaram contradições entre a atenção universal preconizada pelo sistema de saúde haitiano e a existência e discriminações de inequidades, atestadas pela precariedade na atenção à saúde das mulheres.

Embora este estudo tenha tido dimensões pequenas, incluiu todas as regiões sanitárias do país. Ainda, vale ressaltar que a pesquisa foi realizada em apenas uma unidade de atenção primária em cada departamento sanitário. Embora os resultados não possam ser generalizados para o país, os achados são similares aos de outras investigações (26, 30) e contribuíram com a inclusão de gênero e equidade na discussão sobre a atenção a mulheres na atenção primária de saúde no país.

Esta pesquisa constitui uma aproximação entre os estudos de gênero e a avaliação da equidade na atenção à saúde das mulheres. Reforça a premissa de que é preciso usar a categoria gênero como ferramenta transversal nas ações de saúde, buscando reduzir as desigualdades evitáveis e injustas sofridas pelas mulheres e atentando para as vulnerabilidades que incidem sobre elas. Espera-se que esta discussão possa fortalecer a equidade de gênero na atenção à saúde das mulheres tanto no Haiti quanto no contexto latino-americano e caribenho.

### Agradecimentos.

Pesquisa financiada pelo Edital Universal CNPq/2012, projeto 433160/2012-1.

### Declaração de responsabilidade.

A responsabilidade pelas opiniões expressas neste manuscrito é estritamente dos autores e não reflete necessariamente as opiniões ou políticas da *RPSP/PAJPH* nem da OPAS.
